# Molecular Abnormalities and Associated Clinical Features in Polycythemia Vera

**DOI:** 10.3390/medsci14050552

**Published:** 2026-09-08

**Authors:** Ugo Testa, Germana Castelli, Elvira Pelosi

**Affiliations:** Department of Oncology, Istituto Superiore di Sanità, Viale Regina Elena 299, 00161 Rome, Italy; germana.castelli@iss.it (G.C.); elvirapelosi56@gmail.com (E.P.)

**Keywords:** myeloproliferative neoplasms, polycythemia vera, *JAK2*, next-generation sequencing, thrombosis, clonal hematopoiesis

## Abstract

**Background/Objectives**: Myeloproliferative neoplasms are a group of clonal myeloid malignancies that affect bone marrow and include polycythemia vera (PV), essential thrombocythemia (ET) and primary myelofibrosis (PMF). PV is caused in most patients by the *JAK2-V617F* mutation and is characterized at the phenotypic level by overproduction and accumulation of red blood cells. PV is associated with significant morbidity, including risk of thrombotic events and of hematologic evolution (myelofibrotic or leukemic transformation) and reduced survival. In addition to the *JAK2-V617F* mutation, PV patients display additional molecular abnormalities. The aim of this study is to review recent studies investigating molecular abnormalities observed in PV. **Methods**: An extensive search of the most recent literature was performed, selecting and critically analyzing the most relevant studies. **Results**: The studies carried out in recent years have provided an extensive molecular analysis of PV, showing its heterogeneity, characterized in many patients by the presence of additional cytogenetic and gene mutations that contribute to the disease development and evolution. **Conclusions**: PV is a complex disease that needs to be carefully characterized at the molecular level at diagnosis, to be monitored in time to predict the risk for thrombotic complications and hematologic evolution and to receive an adequate treatment.

## 1. Introduction

Myeloproliferative neoplasms (MPNs) are malignant clonal diseases of the hematopoietic system, characterized by the excessive production of mature blood cells. Three main types of BCR-ABL negative MPNs exist: polycythemia vera (PV), essential thrombocythemia (ET) and myelofibrosis (MF). These three MPNs are caused by gain-of-function mutations in one of three different genes: *JAK2*, *CALR* and *MPL*. In particular, *JAK2-V617F* is present in > 95% of PV patients and in about half of ET and MF patients, while CALR and MPL mutations are principally associated with ET and PMF; rare ET and PMF patients are triple-negative [1,2,3]. Approximately half of these patients develop homozygosity for the *JAK2* mutation, due to uniparental disomy on chromosome 9p, which is associated with a higher disease burden. The remaining 2–5% of PV patients carry mutations located to exon 12 of the *JAK2* gene.

**Figure 1:**
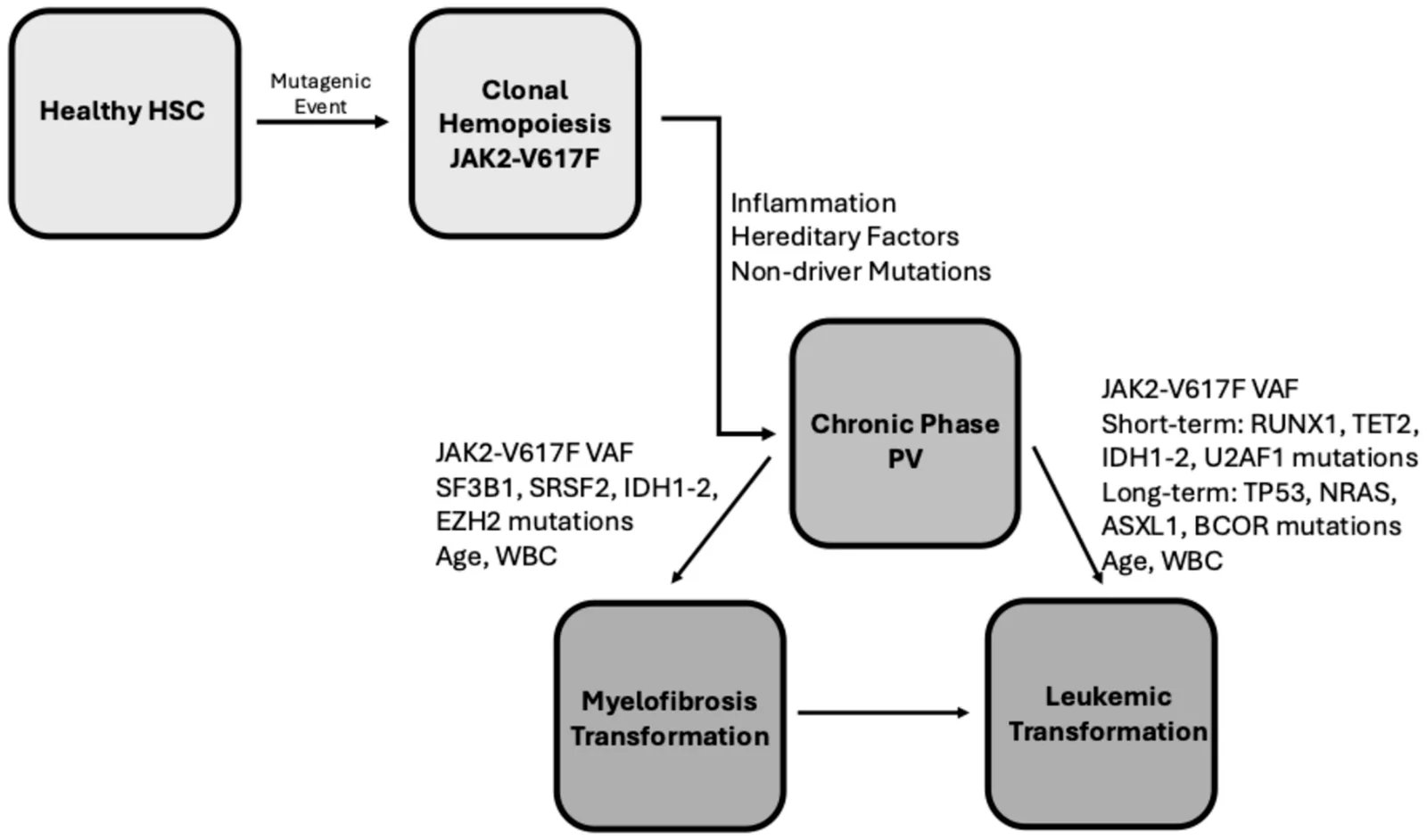
Typical natural history of PV subdivided into three main phases: an initial phase characterized by a mutagenic event occurring in HSCs and causing the occurrence of a *JAK2-V617F* mutation; the expansion of the *JAK2-V617F*-mutant clone is favored by inflammation, non-driver mutations and in some instances by hereditary factors, and determines the progressive development of a clinically detectable PV, mainly characterized by overproduction of RBCs; the chronic phase of PV may have a long duration up to 30–35 years; in 10–20% of patients, PV may undergo a hematologic transformation to myelofibrosis or to acute myeloid leukemia.

PV was first described by a French physician, Louis Henri Vaquez, in 1892. The disease is characterized by overproduction of red blood cells (RBCs). The typical natural history of PV progresses through distinct phases: a preclinical phase initiated by the occurrence of a *JAK2-V617F* mutation at the level of a hemopoietic stem cell; this initial step is followed by a phase of expansion of the mutated clone which requires several decades and leads to the development of the clinically detectable PV disease; a chronic clinical phase, whose duration may arrive up to 30–35 years later, if not interrupted by life-threating thrombotic events; and in 10–20% of patients, the disease may undergo a hematologic transformation leading to myelofibrosis or acute leukemia transformation (Figure 1).

## 2. Aims and Methodology

This narrative review is based on an analysis of the current literature on polycythemia vera. The aim of the present review paper was to critically analyze and discuss recent studies on its molecular characterization and their implications for the clinical phenotype of PV patients.

This study was based on a literature search of the PubMed database, using the terms PV, myeloproliferative neoplasms, PV and venous thrombosis, PV and arterial thrombosis, secondary myelofibrosis, myelofibrotic transformation, and PV and leukemic evolution. The search period extended from 1990 to 2026. The inclusion criteria for the screened literature were limited to studies published in peer-reviewed publications in journals in the English language, in hematology. This review includes the analysis and discussion of experimental and clinical studies in PV, including animal experimental studies and congress abstracts. Only clinical studies based on large cohorts of patients were included.

The Google search engine was used to find the link to major recent international hematology and oncology meetings (American Society of Hematology, American Society of Oncology and European Hematology Association), which were screened for abstracts related to PV.

## 3. Initiating and Driver Alterations

The *JAK2-V617F* mutation causes an oncogenetic program involving a structural and biochemical cascade, determining the shift in JAK2 protein in a constitutively active state. The *JAK2-V617F* mutation consists in a single nucleotide substitution (G⟶T) at position 1849, replacing the amino acid valine (V) with phenylalanine (F) at codon 617 [1,2,3]. This mutation determines a loss of autoinhibition: normal JAK2 protein contains an active tyrosine kinase domain (JH1) and an adjacent, catalytically inactive pseudo-kinase domain (JH2); the JH2 domain acts as an autoinhibitory brake, keeping baseline kinase activity low in the absence of stimulation by cytokines; the *JAK2-V617F* mutation destabilizes the JH2 domain, breaking the autoinhibitory activity over the kinase domain JH1 [1,2,3]. At the structural level, the mutant phenylalanine at position 617 causes strong π-π ring stacking interactions with other aromatic residues (such as F594 and F595 in the αC helix), thus stabilizing an active dimer conformation [4]. The functional consequence of the loss of autoinhibitory control of JAK2 activity is that JAK2 is constitutively activated and no longer strictly requires the binding of an extracellular ligand, such as erythropoietin (Epo) or thrombopoietin (Tpo) to initiate cell signaling [1,2,3]. The constitutive JAK2 activation determines the phosphorylation of its targets: in particular, hyperactive JAK2 continuously phosphorylates the cytoplasmic tails of cytokine receptors, with consequent recruitment and activation of signaling cascades, such as STAT, PI3K/AKT and RAS/MAPK. The continuous JAK-STAT pathway activation promotes uncontrolled proliferation and survival of myeloid and megakaryocyte progenitor cells; the MAPK and PI3K/AKT pathway further stimulates cell cycle progression and promotes cell survival by inhibiting apoptosis [5].

The molecular and biochemical mechanisms triggered by *JAK2-V617F* are responsible for several important clinical consequences that characterize MPN diseases. A first direct consequence that is clinically relevant is that *JAK2-V617F*-mutated stem cells gain a marked survival and proliferation advantage, leading to the abnormal accumulation of RBCs or thrombocytes. A second clinically relevant consequence is that mutant cells produce abnormal levels of proinflammatory cytokines (IL-6, TNF-α, IFN-α), contributing to the generation of an inflammatory microenvironment in the bone marrow that sustains tumor growth and symptom burden (fatigue, weight loss, bone marrow fibrosis) [6]. Furthermore, the *JAK2-V617F* mutation promotes thrombotic and vascular defects related to multiple mechanisms, involving increased clothing activity due to hyperactive platelets, hyperviscosity related in PV to overabundance of RBCs and to endothelial dysfunction promoting abnormal interaction between RBCs and the inner lining of blood vessels.

*JAK2-V617F* is a disease-driver in that it is a causative event that can alone initiate, promote and maintain MPN disease without the need for additional cooperating mutations [7]. However, *JAK2-V617F* is also a clonal driver, in that it can be a marker of clonal hematopoiesis (CH), corresponding to a premalignant condition. In fact, Cordua et al. evaluated the frequency of individuals with *JAK2-V617F* mutations in a cohort of 19,958 individuals of the Danish population using a highly sensitive PCR method with a variant allele frequency (VAF) of 0.01%; on 19,958 probands, 613 (3.1%) carried *JAK2-V617F* and only 16 cases of previously unrecognized MPN were detected [8]. However, the large majority of individuals positive for *JAK2-V617F* mutation did not fulfill diagnostic criteria for MPN [8]. The rate of evolution from *JAK2-V617F* CH to MPN remains to be determined; however, it was evident that individuals with *JAK2-V617F* CH with a VAF > 2% or individuals showing a clear increase in *JAK2-V617F* VAF in the follow-up display a high rate of conversion from CH to MPN [9]. A longitudinal study of blood donors who progressed to MPN suggested that the time required for the conversion to MPN can be estimated in the range of 5–15 years [10]. Whole-genome sequencing studies of single-cell colonies was applied for a reconstruction of the phylogeny and timing of acquisition of the *JAK2-V617F* mutation during the development of MPNs. These studies reached the conclusion that the *JAK2-V617F* mutation occurred decades before the diagnosis of MPN [11,12].

The conversion of CH to MPN is a multi-step process; while the acquisition of driver mutations, such as *JAK2-V617F*, initiates CH, several biological, environmental and genetic factors favor the expansion of these mutant stem cells into clinical MPN.

Among the genetic factors, some factors are of hereditary origin (germline) and play a role selecting the individuals more prone to convert from CH to MPN. Among these hereditary factors, one is represented by familial predisposition: in fact, individuals with first-degree relatives with an MPN disease have a 5–7 times higher risk of acquiring the disease themselves [13]. JAK2 46/1 haplotype is a strong hereditary risk factor of MPN, increasing by two times the risk of developing MPN and representing a significant fraction of the genetic predisposition to MPN [13]. Some hereditary variants, such as those involving *CHEK2* and *GFI1B* loci, favor the CH-to-MPN conversion by increasing the fitness of the mutated stem cells, affecting their proliferation and their self-renewal and function [14]. Inherited variations in the *TERT* gene also increase predisposition to MPN. Finally, certain inherited immune system factors and germline variants modulate the hematopoietic microenvironment, contributing to whether mutated stem cells are recognized and eliminated by the body, or allowed to expand into an MPN.

A second genetic mechanism is represented by non-driver gene mutations cooperating with driver gene mutation and favoring the expansion of mutant *JAK2-V617F* stem cells. In fact, NGS studies have shown that PV at diagnosis exhibits a mean number of 6.5 somatic gene mutations, although a part of these mutations is made up of “passenger” mutations. Additional non-driver mutations may be acquired through two different cellular steps: either after the initial *JAK2-V617F* driver mutation by the acquisition of additional mutations in a branching evolution process defining clones that diverge at several steps or before *JAK2-V617F* driver mutation since the driver mutation is acquired in a preleukemic clone (CH) that already carries a mutation in genes such as *TET2*, *DNMT3A*, *ASXL1*, *SRSF2*, and *SF3B1* [15]. Non-driver mutations mainly pertain to three groups: epigenetic regulation, RNA splicing and tumor suppression. Non-driver gene mutations follow a temporal hierarchy during clonal evolution of PV. Early events mainly involve epigenetic regulators such as *TET2* and *DNMT3A*, and contribute to destabilizing the genome and to promoting self-renewal of mutant HSCs; intermediate-stage mutations, mainly represented by *ASXL1*, *SRSF2* or *SF3B1*, contribute to the development of MPN clones and influence the phenotype of MPN cells; late mutational events, mainly represented by *TP53* or *RAS* mutations, impair the DNA repair mechanism or activate MAPK (PI3K signaling), favoring the leukemic transformation [15].

Inflammatory mechanisms are important modulators of the expansion of mutated hematopoietic stem cells. Experimental models of MPN disease in mice have supported the important role of inflammatory mechanisms in early stages of MPN development. Combining *JAK2-V617F*-induced MPN and knockout of either IL-1β or IL-1R1, Rai et al. showed that both IL-1β and IL-1R1 are required for optimal MPN disease initiation and expansion; furthermore, in vivo inhibition of IL-1β using a neutralizing antibody reduced disease initiation in *JAK2-V617F*-induced MPN mice [16]. In these mice, megakaryocytes and monocytes of *JAK2-V617F*-expressing mice produce more IL-1β than in WT-JAK2 mice [16].

A recent study showed that two healthy lifestyle behaviors, such as uninterrupted sleep and exercise, exert a significant role in *JAK2F-V617* CH clone expansion [17]. These studies were based on the analysis of *JAK2-V617F*-mutant mice. Thus, in atherogenic mice with *JAK2-V617F* or *TET2* mutation, sleep or exercise reduce CH clone expansion [17]. In CH *JAK2-V617F* mutation, sleep or exercise reduce clone expansion by reprogramming mutant, but not concomitant, WT, HSCs and HPCs towards an antiproliferative phenotype by reducing bone marrow macrophage-HPCs IL-1β signaling [17]. In *JAK2-V617*-mutant mice, sleep and exercise reduce atherosclerosis development through an inhibitory activity on the inflammasome activity of arterial macrophages [17].

Finally, the last mechanism that may contribute to favoring clonal MPN expansion is represented by immune system evasion; in particular, clonal evolution into MPN is markedly favored when mutated cells acquire mechanisms to evade immunosurveillance, allowing them to expand without being suppressed by T cells or natural killer (NK) cells.

## 4. *JAK2-V617* Allelic Burden and Associated Clinical Features

The *JAK2-V617* variant allele frequency (VAF), also known as allelic burden, represents the proportion of sequencing reads or detects alleles carrying the mutant allele. A heterozygous *JAK2-V617F* clone involving most nucleated cells may produce a VAF approaching 50%, while VAF above 50% suggests loss of heterozygosity/copy neutral loss of heterozygosity in at least part of the clone. The evaluation of *JAK2-V617F* VAF at diagnosis and during the course of the disease is important because, in PV patients, a VAF > 50% is generally associated with a higher risk of venous thrombosis, larger splenomegaly, and potential progression to myelofibrosis. In PV patients, a low VAF (<10%) can be observed in early stages or as an incidental finding in the context of detection of CH; a moderate VAF (10–50%) is typically observed at the time of PV diagnosis; a high VAF (>50%) is usually observed in patients with longer disease duration and implies that the mutated cells become dominant over the normal cells. Stem cell fitness in PV is correlated with *JAK2-V617* VAF in HSC and progenitor cell compartments, with higher levels in these compartments being associated with higher fitness levels [18]. Evaluation of *JAK2-V617* VAF in peripheral blood is an inappropriate marker in PV for evaluating HSC fitness. However, in spite these limitations, at a clinical level, a high *JAK2-V617* VAF in PB is an established risk factor for vascular complications and for progression to post-PV myelofibrosis [19,20]. A PB *JAK2-V617* VAF > 50% is an increased risk factor for venous thrombosis even after adjusting for prior events, WBC, and age [21]. Guglielmelli et al., through the study of a large cohort of 576 PV patients, provided evidence that *JAK2-V617* VAF >50% was a strong predictor of venous thrombosis; this conclusion was observed both in low-risk and high-risk PV patients [22]. Importantly, this study showed that *JAK2-V617F* VAF > 50% represents an increased risk for venous thrombosis but not for arterial thrombosis; in these patients, the presence of diabetes, hyperlipidemia and previous arterial thrombosis are independent risk factors for arterial thrombosis.

A meta-analysis, including 21 studies, explored the association of *JAK2-V617F* VAF with clinical correlates in PV patients [23]. This analysis showed that patients who had higher *JAK2-V617F* VAF had a significantly increased tendency for developing pruritus, splenomegaly, thrombosis, myelofibrosis, and AML [23]. Furthermore, leukocyte and hematocrit counts were significantly higher in patients with higher *JAK2-V617F* VAF [23].

In conclusion, these studies support the important role of *JAK2-V617F* VAF in the hematologic evolution of PV disease and in the development of thrombotic events; therefore, *JAK2-V617F* VAF evaluation at diagnosis and its monitoring during the course of disease is highly recommended.

## 5. Cytogenetic Abnormalities in PV

Approximately 15–20% PV patients at diagnosis exhibit abnormal karyotypes, with the frequency rising over time and during fibrotic or leukemic transformation. The most common chromosomal alterations observed in PV are trisomy 9 (+9), loss of the Y chromosome in males (-Y), deletion of the long arm of chromosome 20 (del(20q)) and trisomy 8 (+8). In addition to the canonical driving *JAK2-V617F* mutation, karyotypic abnormalities may represent a molecular mechanism triggering progression of the disease clone.

Many studies have investigated the role of chromosomal abnormalities (CAs) in PV progression. An initial study by Swolin and coworkers reported a longitudinal analysis in 64 PV patients: at initial observation, 11 (17%) patients had chromosomal abnormalities, and 20 additional patients developed CAs during the course of disease; an abnormal karyotype (AK) was observed in 71% to 80% of patients after development of myeloid metaplasia, myelofibrosis or leukemia [24]. Patients treated with myelosuppressive agents showed a significantly greater risk of CAs than did patients who had been phlebotomized [24]. Surprisingly, in this study, no consistent relationship was observed between the initial presence of CAs and the development of myelofibrosis or leukemia [24]. A similar conclusion was reached in another early study by Gangat et al., reporting CAs in 15 of 137 PV patients at diagnosis: -Y in 7% of male patients and other chromosomal abnormalities (+8, +9, del(20q)) in 11% of patients [25]. Age but not *JAK2-V617F* allele burden and fibrotic or leukemic transformation were associated with CAs [25]. In 2013, a large international study on 1545 PV patients provided evidence that older age, AC at diagnosis and leukocytes > 15 × 10^9^/L are associated with reduced survival and increased risk of leukemic transformation [26].

**Figure 2:**
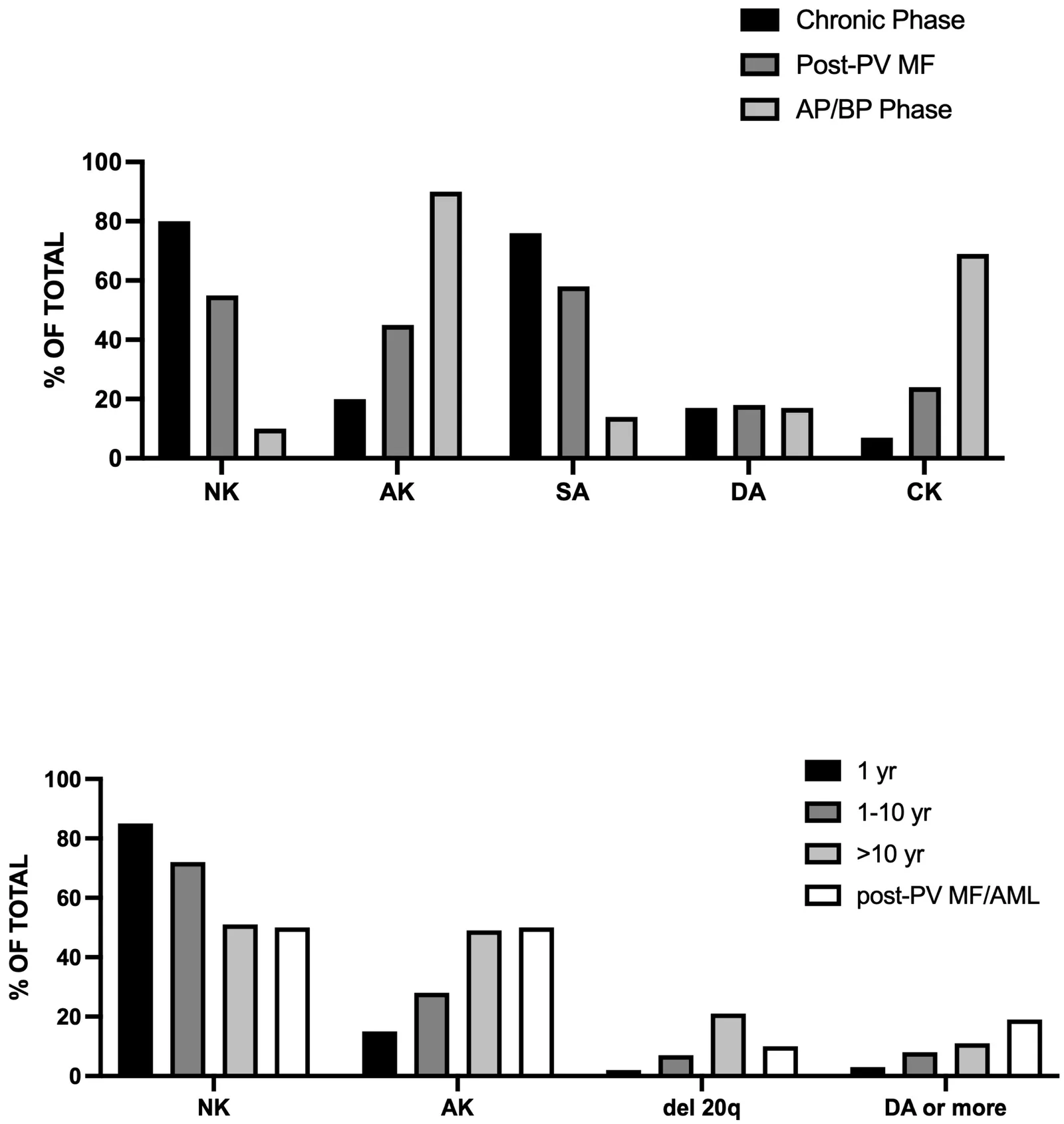
Chromosomal abnormalities in PV. **Top Panel**: Chromosomal abnormalities in PV patients at different stages of disease: chronic phase, post-MF and post-leukemic transformation. Original data are reported in Tang et al., 2018 [27]. **Bottom Panel**: Chromosomal abnormalities in PV patients explored in the chronic phase either within 1 year (1 yr), 1–10 years (1–10 yr) or >10 years (>10 yr) from diagnosis or post-MF/AML transformation. Original data reported in Iftikhar et al., 2025 [29]. Abbreviations: NK (normal karyotype), AK (abnormal karyotype), SA (single chromosomal abnormality), DA (double chromosomal abnormality) and CK (complex karyotype).

In 2017, Tang et al. provided an extensive analysis of characteristics and clinical significance of CAs in a large cohort of PV patients, including 271 patients in the polycythemic phase (PP), 112 post-PP myelofibrosis (PPP), 11 in the accelerated phase and 28 in the blast phase [27]. The frequency of CAs greatly changed in these various stages of PV: 20% in PP, 45% in post-PP MF, 90% in the accelerated/blastic phase [27]. Similarly, the types of CAs also changed in the different stages of PV: single abnormalities including 20(q), +9 and +8 were the most frequent CAs observed in PP patients; single CAs were less frequent MF patients, with rare-absent +8 and +9 and with the presence of 24% of complex karyotype (CK) (with chromosome 5, 7 and 17 abnormalities); single CAs were virtually absent in the accelerated/blastic phase, with predominant CK (69%) with its typical CAs [27] (Figure 2).

Patients in chronic PP received one, two or more evaluations for the cytogenetic profile: (i) 146 PP patients with multiple BM evaluations showed disease progression to post-PP MF (31 patients) and to the accelerated/blastic phase (14 patients): patients with AK had a higher risk of disease progression compared to those without CAs; (ii) 59 PP patients had BM evaluation only at diagnosis and patients with AK had a higher frequency of transformation (60% vs. 10%) and a shorter transformation survival (101 months vs. undefined) compared with patients with normal karyotype; (iii) in 76 patients with post-PP MF, the presence of CAs shortened transformation-free survival but did not modify the risk of transformation compared to patients without CAs [27]. The presence of AK had a negative impact in OS in both PP and post-PP MF patients; concerning the presence of single CAs, only del(20q) had a negative impact on OS in PP patients; the presence of CK in both PP and post-PP MF had a markedly negative effect on OS [27]. The acquisition of additional CAs was more pronounced among patients with AK compared to those with normal karyotype and was associated with increased tendency to disease progression [27]. According to all the data obtained, prognostic risk evaluation of cytogenetic abnormalities was elaborated, with the identification of three risk groups with distinctly different OS profiles: low-risk group, including patients with normal karyotype and sole +8, +9 abnormalities; intermediate-risk, including patients with sole del(20q) and double chromosome abnormalities; and high-risk, including patients with CK [27].

In 2017, Barraco et al. provided a more extensive analysis of CAs in 196 PV patients: 19% had AK, 4% for unfavorable karyotype, 17% for sole abnormalities and 0% for complex karyotype [28]. In univariate analysis, AK adversely affected OS, LFS and MFFS (myelofibrosis-free survival), but not TFS (thrombosis-free survival); inferior outcome was observed also for unfavorable karyotype [28]. On multivariate analysis for OS, which included age, leukocyte count, thrombosis and adverse mutations, the detrimental effect of AK and loss of Y remained significant [28].

Iftikhar et al. reported an extensive characterization of the CAs observed in 669 PV patients: 436 evaluated within 1 year from diagnosis, 116 within 1–10 years, 47 after more than 10 years and 70 at post-MF or leukemia transformation (LT) [29]. The frequency of CAs progressively increased in patients with disease duration from 15% to 28%, 49% and 50–67%, respectively, in the four groups of patients described above [29] (Figure 2). In patients evaluated within 1 year from diagnosis, −7 (3%), +9 (3%), del(20q) (2%), +8 (1%) and ≥2 abnormalities were the most frequent CAs; the frequency of del(20q) and single abnormalities excluding del(20q), +8, +9. -Y, and ≥2 abnormalities including CK markedly increased with longer disease duration [29]. The analysis of patients studied 2–3 times for their cytogenetic profile post-diagnosis provided evidence of clonal evolution both during chronic and transformation phases [29]. AK correlated with older age, lower platelet count and grade ≥ 2 reticulin fibrosis. At a median follow-up of 7.4 years, 37% of death, 11% of fibrotic transformation and 3% of leukemic transformation were documented [29]. In univariate and multivariable analyses, AK was associated with a reduced OS and with an increased risk of developing post-PV MF and LT [29].

Tefferi and coworkers explored the cytogenetic clonal evolution in a cohort of 648 patients with MPN (9% with ET, 26% with PV and 28% with PMF) explored with serial BM biopsies [30]. Baseline karyotype was abnormal in 9% of ET patients, 14% PV patients and 40% of PMF patients; new cytogenetic abnormalities were detected in 14% of patients with ET, 25% with PV and 32% with PMF [30]. A change in karyotype from normal to abnormal, in the absence of overt disease transformation into fibrotic or blast phase disease, showed a trend of adverse survival in both ET and PV patients and a reduced survival in PMF patients [30].

As discussed above, in PV, an acquired trisomy 8 is one of the most recurrent numerical chromosomal abnormalities, being observed in 1–3% of patients at diagnosis. An additional chromosome 8 copy can be acquired during disease course, an event that can be associated with adverse outcomes. Trisomy 8 is frequently observed in cases of PV that have transformed to MF or AML. Trisomy 8 in myeloid neoplasms is known to drive the overexpression of the MYC gene, located at 8q24. A recent study showed that trisomy 8 causes *MYC* overexpression and may contribute to driving a peculiar subtype of MPN in triple-negative MF patients [31].

As discussed above, chromosome 9 trisomy or partial gains on the short arm 9p is a recurrent CA in PV, observed in 2–3% of cases at diagnosis. Trisomy alone is not conclusively recognized as an independent prognostic factor in PV patients in the early stages. Trisomy 9 results in three copies of all gene loci in the affected chromosome arm; for patients with the *JAK2-V617F* mutation, this frequently translates to having two mutated *JAK2* alleles and one unmutated allele, thus accelerating oncogenic signaling. Trisomy 9 acts as a disease modifier in *JAK2-V617F* patients; in fact, CD34^+^ cells isolated from *JAK2-V617F* patients with trisomy 9 displayed increased clonogenicity, generating in vitro an increased number of immature colonies [32]. Furthermore, *JAK2-V617F*-mutant patients showed increased PD-L1 expression, a condition that fosters T-cell exhaustion and favors growth of the malignant clone through immune evasion [32]. At the level of chromosome 9, another frequent chromosome abnormality is represented by chromosome 9p loss of heterozygosity (9p-LOH). The *JAK2-V617F* mutation occurs in a single allele present on chromosome 9; during cell division, a mitotic recombination can occur, resulting in a LOH, where the cell retains two copies of chromosome 9, but both copies carry the mutant *JAK2* allele, not visible on standard cytogenetic analysis because the total number of chromosomes remains unchanged, but is detectable using SNP array technology.

Recent studies suggest that 9p LOH plays a role as a factor favoring PV transformation to MF. In a large genomic characterization of 2035 MPN patients, including 356 PV patients, Grinfeld at al. reported the classification of seven genomic groups of patients subdivided according to the type of driver mutations; two groups were characterized by *JAK2-V617F* mutations and were subdivided into a group with homozygous *JAK2* mutation (due to 9p LOH) and a group with heterozygous *JAK2* mutation [33]. In the group of homozygous *JAK2-V617F* mutation, PV patients were the most frequent patients, while ET patients were rare; in contrast, in the group of heterozygous *JAK2-V617F* patients, both ET and PV patients were the most frequent [33]. Among PV patients, about half of patients were homozygous and half were heterozygous for *JAK2-V617F* mutation. The group of *JAK2-V617F* homozygous PV patients displayed a higher rate of MF and leukemic transformation compared to the *JAK2-V617F* heterozygous patients [33]. Oh et al. have evaluated the role of *JAK2-V617F* and 9p-LOH in the transformation to MF in PV patients, in the context of the REVEAL study [34]. A total of 454 patients were explored in this study: 62% had 9p-LOH, 3% had trisomy 9 and 30% had no chromosome 9 abnormalities; a *JAK2-V617F* > 50% was predictive of 9p-LOH in 100% of cases; however, a 9p-LOH was not necessarily associated with *JAK2-V617F* VAF > 50%; 9p-LOH but not trisomy 9 was predictive of MF transformation [34]. A *JAK2-V617F* VAF > 75% was highly predictive of MF transformation [34].

As discussed above, a 20q deletion (del(20q)) is a common cytogenetic abnormality in PV patients, occurring in about 8–10% of cases at diagnosis. It arises in early HSCs and is linked to older age and increased marrow fibrosis. The deletion usually involves the long arm of chromosome 20 and occurs as an interstitial deletion, leading to the loss of potential tumor suppressor genes that confer a proliferative advantage to myeloid cells. Its presence correlates with a higher risk of developing MF.

## 6. Additional Somatic Mutations in PV

About 50% of PV patients carry additional non-driver mutations; these mutations do not cause the development of PV, but influence the risk of thrombosis, disease severity and likelihood of progression to MF or leukemia. PV patients may acquire additional mutations in genes regulating epigenetics, RNA splicing and DNA repair.

**Figure 3:**
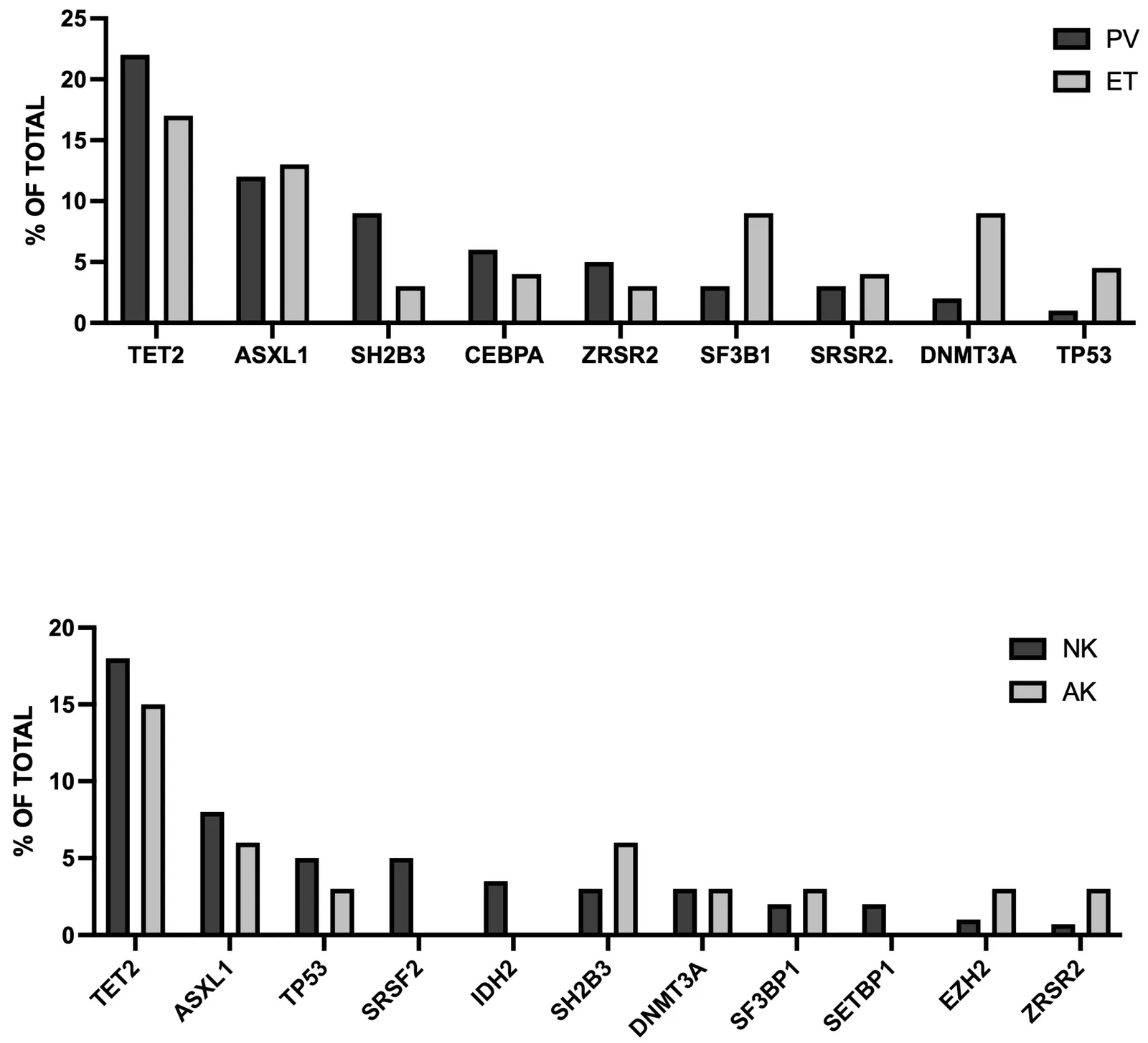
Non-driver gene mutations in PV and ET patients. **Top Panel**: Comparison of the frequency of the main non-driver gene mutations in PV and ET patients. Original data are reported in Tefferi et al., 2016 [35]. **Bottom Panel**: Comparison of the frequency of the most recurrent non-driver gene mutations in PV patients with normal (NK) or abnormal karyotype (AK). Original data are reported in Iftikhar et al., 2025 [29].

A key study in 2016 reported the results of targeted deep sequencing in a cohort of 316 Mayo Clinic patients with PV (133 patients) or ET (183 patients) with a long-term evaluation of their survival [35]. As expected, 98% of PV patients had canonical JAK2 driver mutation, while ET patients had *JAK2* (52%), *CALR* (26%) or *MPL* (4%) driver mutations [35]. The mutational profile of non-driver gene mutations was similar in PV and ET patients; only *SF3B1*, *TP53* and *DNAMT3A* were more frequent among ET than PV patients, while *SH2B3* mutations were more frequent in PV than in ET patients [35] (Figure 3).

In both groups of patients, *TET2* and *ASXL1* were the most recurrent non-driver mutations; in ET patients, *ASXL1* co-segregated with *EZH2*, *IDH2* and *RUNX1* mutations and in PV with *IDH2* and *KIT* mutations; *TET2* in PV co-segregated with *SH2B3* [35]. Some mutations displayed remarkable phenotypic associations: *TET2* mutations were associated with thrombosis in ET patients; in ET patients, *TET2* and *SFB1* mutations associated with older age; *ASXL1* and *SH2B3* mutations associated with palpable splenomegaly in ET and PV patients [35]. The analysis of prognostic impact showed that, in PV patients, adverse mutations included *ASXL1*, *SRSF2*, and *IDH2*: these mutations, with a combined prevalence of 15%, were associated with inferior OS (7.7 vs. 16.9 years) and the effect was independent of conventional prognostic models [35].

Morishita and coworkers analyzed non-driver gene mutations in a group of 246 PV and 579 ET patients and confirmed that the mutational profile was highly comparable in the two groups of patients [36]. Furthermore, as well as in the study of Tefferi et al. [35], they observed a higher frequency of *SF3B1* mutations in ET than in PV patients (2.6% vs. 1.1%, respectively) and a trend toward higher *ASXL1* mutational frequency in PV than in ET patients (6.8% vs. 3.8%, respectively) [36]. In both PV and ET patients, *ASXL1* mutations were found as a risk factor for leukemic/myelofibrotic transformation [36].

Senin and coworkers have explored non-driver mutations in a cohort of 100 patients with *JAK2-V617F*-mutated PV or ET with long-term molecular follow-up [36]. The most frequently mutated genes were *TET2*, *DNMT3A*, *TP53* and *ASXL1*; the VAF of most of the mutated genes expanded during the follow-up, with a significant increase being observed for *TET2* and *ASXL1* [37]. The presence of non-driver mutations at diagnosis was associated with an increased risk for acquiring new genetic events [37]. Patients with additional mutations at first evaluation post-diagnosis had a higher probability of developing cytopenia under hydroxyurea cytoreductive therapy; patients with *ASXL1*, *TP53*, *SRSF2*, *IDH1/2* and *RUNX1* have an increased probability of leukemic transformation [37]. The paired analysis of non-driver gene mutations in *JAK2-V617F* mutated PV and ET patients before and after MF or leukemic transformation showed that MF transformation did not significantly change the mutational profiles of non-driver mutations, while the leukemic transformation was associated with a significant increase in *TET2*, *TP53*, *RUNX1*, *SF3B1*, *SETBP1*, *SH2B3*, *CBL* and *IDH1/2* mutations clustered with normal karyotype [37].

The inclusion of information relative to non-driver mutations enhanced the prognostic value of international systems for PV ET evaluation [38]. In particular, for PV, age > 67 years, leukocytosis > 15 × 10^9^/L, thrombosis history and prevalence of *SRSF2* mutations predicted reduced survival [37]. Kandula and coworkers explored a group of 151 PV patients and observed non-driver mutations in 53% of cases, mainly involving *ASXL1*, *SRSF2*, *U2AF1* and *IDH2* [39]. MF transformation in these PV patients was affected by *JAK2-V617F* VAF and by the presence of coexisting non-driver mutations [39].

The comparison of the mutational profile of non-driver gene mutations in PV patients with normal karyotype or with abnormal karyotype showed some remarkable difference in that *SFRSF2*, *IDH2* and *SEBP1* mutations clustered with normal karyotype [29] (Figure 3).

A recent study reported an extensive characterization of non-driver mutations in a cohort of 439 PV patients. Non-driver mutations were detected in 53.3% of patients, of whom 13.4% had two mutations and 9.3% had three or more mutations [40]. The most frequently detected mutations involved *TET2*, *ASXL1*, *NF-E2*, *PPM1D* (mostly truncating mutations), *DNMT3A*, *CBL*, *SRSF2*, *IDH1/2*, *SF3B1* and *TP53* (mostly missense mutations) [40]. The VAF was variable for different mutated genes, being low for *DNMT3A* and *NF-E2* mutations, but usually high for *IDH2* and *SRSF2*; *TET2* and *ASXL1* had a bimodal VAF distribution with a group at low VAF and a group with VAF around 40% [39]. Phenotypic associations showed that *TET2*, *DNMT3A*, *ASXL1* and *SRSF2* mutations were associated with older age; *ASXL1*, *SRSF”* and *IDH1/2* mutations were associated with higher monocyte counts [40]. A Bayesian network analysis showed that *ASXL1* mutations represent a central node in the molecular landscape, being isolated in only 22% of cases, and in the rest of the cases, are associated with mutations in *TET2*, *EZH2*, *SRSF2* or *IDH1/2* genes [40]. *TET2* (missense and truncating mutations), *SRFSF2*, *IDH1/2*, *EZH2* and *ASXL1* mutations were associated with reduced OS; *NFE2*, *SRSF2*, *EZH2* mutations and *SRFSF2*, *IDH1/2* mutations were associated with secondary myelofibrosis and AML transformation, respectively [40]. Furthermore, the total number (two or more) of non-driver gene mutations had a negative impact on OS and transformation-free survival [40]. According to these observations, a genomic classification was developed predicting OS and TFS better than other scoring systems adopted for PV patients [40].

Mora and coworkers recently evaluated the genetic alterations in PV and ET patients who have developed secondary myelofibrosis, in the context of the MYSEC (myelofibrosis secondary to PV and ET) study [41]. The study was based on the evaluation of 644 patients with secondary MF by NGS; 429 (66.6%) of these patients had at least one non-driver gene mutation; *TET2*, *ASXL1* and *DNMT3A* were the genes most recurrently mutated [41]. Some gene mutation profiles were associated with OS: *U2AF1*, *TP53* or *SRFSF2* variants (UTS, mOS 4.1 years), and *ASXL1* mutations without UTS (mOS 8.4 years) [41]. By integrating these genetic signatures, a MYSEC-Prognostic Model was developed, based on some independent predictors of survival: hemoglobin level < 11g/dL, circulating blasts > 3%, platelets < 150 × 10^9^/L, age, *ASXL1* without UTS and any UTS mutation [41]. The MYSEC-PM classified these patients in four groups: low (mOS 18 years), intermediate-1 (8.8 years), intermediate-2 (4.6 years) and high-risk (1.9 years) [41]. The MYSEC-PM could be further implemented by cytogenetic profile [41].

Rana and coworkers explored the mutational landscape in a large cohort of PV patients (319 patients), subdivided into three groups: group A at diagnosis or in the chronic phase (85%); group B at the time of fibrotic transformation (12%); and group C at the time of leukemic transformation (3%) [42]. Mutational frequencies of *TP53*/*SRSF2*/*IDH1*/*U2AF1* were markedly different in patients of group A (2%/4%/2%/0.4%), group B (8%/0%/0%/5%) and group C (50%/25%/17%/8%) [42].

A detailed analysis of the group A patients showed: *ASXL1* mutations were associated with younger age and leukocytosis; *TP53* mutations were associated with leukocytosis; mutation co-segregation was apparent between *ASXL1* and *IDH2* or *SRSF2*, *SRSF2* and *IDH2*, *TP53* and *NRAS*; and multivariable analysis showed mutations in *SRSF2*, *IDH2*, *ASXL1*, leukocyte count ≥ 15 × 10^9^/L and advanced age as risk factors for OS [42]. Median OS in the absence or in the presence of any adverse mutation (*SRSF2*, *ASXL1*, *IDH2*) was 17.8 vs. 8.8 years, respectively [42]. The number of non-JAK2 mutations was significant in predicting outcome in univariate but not multivariable analysis [42].

As discussed above, *ASXL1* mutations are usually observed in 5–15% of PV patients at diagnosis. When present, *ASXL1* mutations are associated with increased risk of thrombosis, enhanced bone marrow inflammation, and shorter survival. Acquisition of *ASXL1* mutations favors progression to MF. In a large cohort of 1004 PV patients, Zhang et al. showed that the presence of *ASXL1* mutations increases the risk of developing thrombosis [43]. Diaz et al. observed that the increased thrombotic risk was particularly evident in patients with *ASXL1* mutations associated with *TET2* but not with *DNMT3A* mutations [44]. The presence of *ASXL1* mutations accelerates bone marrow fibrosis through a biochemical pathway involving EGR1-TNFα-mediated neoplastic fibrocyte generation [45].

It is of interest to note that the transcription factor NFE2 is frequently altered in PV patients. In fact, NFE2 is a downstream target of activated JAK2 and its expression increased in PV through a stimulatory effect mediated by *JAK2-V617F* [46]. Furthermore, *NFE2* mutations are observed in 7.3% of PV patients [46]. While *NFE2* overexpression is a primary driver event contributing to expansion of erythroid cells in PV, somatic *NFE2* mutations are a secondary genetic event. These mutations, insertions or deletions causing frameshifts, confer a greater proliferative advantage to cells already expressing mutant *JAK2* [46]. *NFE2* mutations in PV are associated with a reduced OS and an increased risk of leukemic transformation [46].

In conclusion, a high percentage of PV patients display somatic genetic alterations (cytogenetic abnormalities and gene mutational events) in addition to the primary driver event at the level of *JAK2* genes. These additional genetic alterations contribute to PV development and to the clinical phenotype of PV patients. Ideally, all PV patients with atypical features and at risk of progression should be screened for the whole spectrum of genetic alterations, which represents key information to assess the risk of hematologic disease evolution and of thrombotic events.

## 7. Molecular Determinants of Fibrotic and Leukemic Transformation

In PV, it is of fundamental importance to identify patients who have a greater risk of hematological disease evolution, in terms of myelofibrotic or leukemic transformation.

### 7.1. Myelofibrotic Transformation

Myelofibrotic transformation of PV, known as post-PV myelofibrosis, occurs in approximately 10–20% of patients. Key factors driving post-PV myelofibrosis include advanced age, prolonged disease duration, elevated WBC counts, a higher *JAK2-V617F* burden, some non-driver mutations and an unstable or complex karyotype. The progression of PV to MF is characterized by changes in bone marrow histology, becoming increasing scarred and fibrotic, which frequently results in declining blood cell counts and an enlarged spleen.

A persistently high or progressively increasing burden of the *JAK2-V617F* mutations (*JAK2-V617F* VAF > 50%) is one of the strongest predictors of fibrotic progression. The mechanism of MF triggered by higher *JAK2-V617F* VAF are related to a greater overactivation of the JAK-Stat signaling pathway, resulting in higher WBC counts and increased secretion of inflammatory cytokines that trigger bone marrow fibrosis. As PV disease progresses, patients often transition from being heterozygous (VAF near 50%) to homozygous (VAF near 100%) for *JAK2-V617F* mutation, due to 9p-LOH. The Prospective Observational Study of Patients with Polycythemia Vera in US Practice Trial (REVAEL) investigated the clonal architecture and molecular mechanisms predicting transformation to myelofibrosis in PV patients [47]. A total of 1880 of the 2510 patients enrolled in the REVEAL study had a detailed molecular analysis; 114 of these 1880 patients had myelofibrosis transformation during the study period [47]. The clinical characteristics of patients at enrollment were similar in the transformed and non-transformed groups. The transformed and non-transformed groups differed for some genetic features: median *JAK2-V617F* VAF was significantly higher in the transformed vs. non-transformed groups (84.2% vs. 57.4%, respectively); a higher number of combined non-driver mutations was observed in the transformed vs. non-transformed group: in particular, *SF3B1*, *IDH-1/2*, *EZH2* and *TP53* mutations were enriched in the transformed group [47]. Furthermore, the VAF of non-driver mutations trended higher in the transformed compared to the non-transformed group [47]. Analysis of CNV provided evidence that inflammatory pathway genes could play a relevant role in MF transformation in PV patients [47]. In a more recent analysis, the longitudinal molecular changes observed in 41 of these 114 PV patients who underwent myelofibrosis transformation were reported [48]. Heterogeneous and distinct molecular patterns were observed in these patients. 9p-LOH was observed in 85% of these 41 patients at enrollment, which correlated with higher *JAK2-V617F* VAF. Of six patients without 9p-LOH at enrollment, five acquired 9p-LOH at myelobibrosis transformation [48]. *JAK2-V617F* increased >5% in 17 patients, remained stable in 13 patients, and decreased >5% in 11 patients at transformation [48]. Additional non-driver gene mutations were identified in 18/41 patients [48]. It is important to note that, in this study, among the four clinical factors identified as possible predictors of disease progression—history of thrombotic events, high WBC, low hematocrit level and age—high WBC count resulted in being the most significant predictor of myelofibrosis transformation [48].

The classical clinical prognostic factors described in PV, such as older age, history of thrombosis, constitutional symptoms (fatigue, sweating or weight loss) were associated with decreased overall survival, but not with the risk of hematologic transformation. The identification of PV patients at high risk of hematologic transformation requires the study of molecular markers. In this context, the study by Monsier et al. showed that *NFE2*, *SRSF2*, *EZH2* and the total number of non-driver mutations (two or more in the same patient) were associated with secondary MF, while *SRSF2*, *IDH1/2* and the total number of non-driver mutations were associated with AML transformation [40]. According to these findings, a molecular prognostic signature was proposed based on the definition of three groups: a PV-HMR (high-molecular-risk) signature englobing patients with mutations in *SRSF2*, *IDH1/2*, *EZH2* and/or *NFE2*, or more than one additional non-driver mutation or presence of at least one non-9p CNV; patients with *TET2* mutations with a VAF > 5% were considered at intermediate risk; other genetic profiles were considered at low risk [40]. Using multistate modeling, evidence was provided that PV-HMR groups and age at diagnosis were associated with an independent and significantly higher risk of hematologic transformation and risk of death without hematologic transformation [40].

In conclusion, myelobibrosis transformation is observed in 10–20% of PV patients, and some clinical and genetic factors are associated with this event. The monitoring of these factors may help to predict the risk of myelofibrotic transformation during the course of PV and the monitoring of patients considered at risk of this event is recommended.

### 7.2. Leukemic Transformation

Evolution to secondary AML is observed in PV and ET patients in 2 to 5% of patients at 15 years post-diagnosis. Primary risk factors of leukemic transformation are traditionally considered older age (>60 years), longer disease duration, elevated WBC counts, abnormal chromosomal profiles and specific high-risk mutations. Historical exposure to alkylating agents and sequential use of multiple myelosuppressive drugs also elevate this risk. The prognosis of AML post-PV or post-ET is poor and is usually associated with a mOS of few months.

As discussed above, the mutational profile of non-driver mutations of post-PV AMLs differs from that observed in the chronic phase [40]. Importantly, the frequency of many mutated genes differs in post-PV AML compared to that typically observed in de novo AML for a markedly lower frequency of *FLT3* and *NPM1* mutations and a markedly higher frequency of *TP53*, *ASXL1*, *TET2* and *RUNX1* mutations [49].

**Figure 4:**
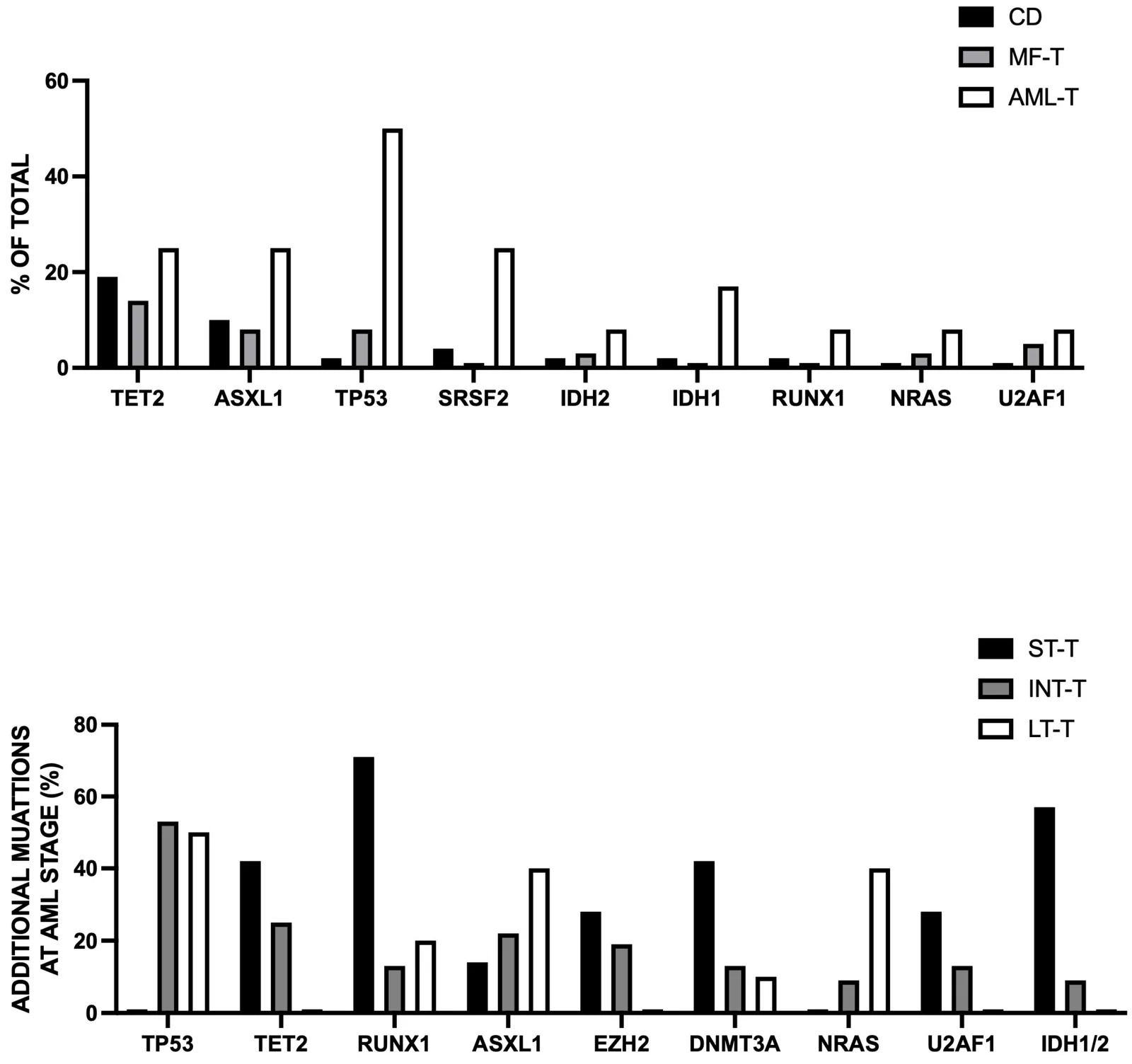
**Top Panel**: Profile of non-driver mutations in PV patients during chronic disease (CD) or after MF (MF-T) or leukemic transformation (AML-T). Original data are reported by Rana and coworkers, 2025 [42]. **Bottom Panel**: Additional non-driver mutations acquired after AML transformation in PV patient exhibiting a short-term (ST-T), intermediate-term (INT-T) or long-term (LT-T) leukemic transformation. Original data reported in Luque Paz et al., 2020 [50].

The interval between diagnosis and leukemic progression is highly variable from one patient to another, ranging from few years to more than 20 years. Few studies have characterized the mutational landscape of PV and ET patients post-AML transformation and have compared it to that observed during the chronic phase. In this context, a key study by Puque Paz and coworkers reported a detailed molecular characterization of 49 PV and ET patients during the chronic phase and post-leukemic transformation. At leukemic transformation, the patients exhibited a median number of four additional non-driver mutations, undetectable during the chronic phase [50]. The most frequently mutated genes both in PV and ET patients were *TP53*, *TET2*, *RUNX1*, *ASXL1*, and *EZH2* [50]. The timing of leukemic transformation was very different, and therefore, these patients were subdivided into three groups: a group with short-term, a group with intermediate-term and a group with long-term transformation time. These three groups consistently differed in the profile of the acquisition of new mutations [50]. Thus, short-term transformations were characterized by mutations in *RUNX1*, *IDH1/2*, *U2AF1*, *DNMT3A* and *TET2* genes, while the long-term transformations were enriched in *TP53*, *NRAS*, *ASXL1* and *BCORL1* gene mutations [50] (Figure 4). These observations have suggested that leukemic transformation of PV and ET may be driven by distinct time-dependent molecular mechanisms [50].

As discussed above, the transformation of PV to sAML is associated with the acquisition of additional chromosomal abnormalities, such as complex karyotype and specific abnormalities, most notably involving chromosomes 1, 5, 7, 8, 9, 17 and 20 [27,29]. Key cytogenetic findings during leukemic transformation include a complex karyotype, defined as ≥3 chromosomal abnormalities, frequently observed in these patients and indicating genomic instability; chromosome 17p deletions (loss of the short arm of chromosome 17), often associated with *TP53* mutations; and chromosome 5 and 7 deletions, the classic markers of sAML and myelodysplastic syndromes.

The treatment of patients with post-PV AML is particularly challenging for the consistent resistance to all the standard treatments used for de novo or s-AML or MPN patients. Interestingly, recent studies have shown that the gene encoding the anti-apoptotic *BCL-X*_L_ protein is expressed at higher levels in post-MPN AML than in de novo AML, thus suggesting a rationale for targeting *BCL-X*_L_ in these patients [51,52]. In line with this suggestion, targeting of BCL-X_L_ together with JAK2 targeting resulted in a significant anti-leukemic effect in primary post-MPN leukemic cells and in mouse models of *JAK2*-mutated post-MPN AML [51,52].

### 7.3. TP53-Mutant PV

*TP53* mutations are observed in a minority of PV patients.

Ultradeep NGS analysis showed that the presence of low-burden *TP53* mutations (0.2% to 11.6% VAF) was detectable in 26.8% of patients; the *TP53* mutations were strongly associated with age [53]. In most patients, these mutations may persist at low levels for years without immediate risk of progression.

Famoud et al. explored *TP53* mutations in a large cohort of MPN patients in the chronic phase and in the leukemic/blastic phase [54]. In patients in the chronic phase, *TP53* mutations were observed in 6%, 13% and 15% of PV, ET and PMF patients, respectively, while *TP53* mutations were detected in 66% of patients in the leukemic/blastic phase [54]. Furthermore, *TP53*-mutant VAF was significantly higher in patients in the leukemic phase compared to those in the chronic phase [54].

Rolles and coworkers have explored a group of 1540 MPN patients and 111 had mutations in the *TP53* gene (32% with PV, 39% with ET and 25% with PMF) [55]. A total of 67% of mutant *TP53* patients were defined by single-hit status (monoallelic disease) and 33% by multi-hit status (biallelic disease) [55]. sAML developed in 1% of patients with a recent diagnosis of *TP53*-WT PV/ET, in 6% of patients with PMF/*TP53*-WT/prefibrotic MF/secondary myelofibrosis and in 23% of patients with *TP53*-mutant MPN [55]. Among patients diagnosed with PV or ET, those with *TP53* mutations had a significantly shorter mOS and mLFS than those without *TP53* mutations (for OS, 16.8 years vs. 29.8 years; for LFS, 16.3 years vs. 29.0 years, respectively) [55]. mOS and mLFS were significantly shorter in *TP53*-mutant patients with multi-hit *TP53* allelic status compared to those with mono-hit *TP53* allelic status (for OS, 0.8 years vs. 3.2 years; for LFS, 0.7 years vs. 2.9 years, respectively) [55]. In multivariable analysis, the presence of fibrosis in bone marrow and multi-hit mutation status were associated with worse OS [55].

In conclusion, leukemic transformation is a rare event observed in a minority of PV patients, associated with a poor outcome. Leukemic transformation may occur at early or late time points during the course of disease, and clinical and genetic factors favoring leukemic transformation must be monitored in patients considered at risk for this type of event.

## 8. Risk of Secondary Non-Hematologic Malignancies in PV

PV patients have a high rate of secondary hematologic and non-hematologic cancers. The incidence of secondary cancers is increased by about 30 to 60% in patients with PV compared with the general population, with reported incidence of PV-associated secondary malignancies ranging from 1.6 to 3.0 per 100 patient-years [56,57]. In particular, an increased incidence of skin, lung, kidney, and thyroid cancers was observed in patients with PV older than 60 years; however, the risk of other solid tumors such as colon, breast and prostate cancer did not significantly differ from that observed in the general population [58].

In a population-based study of nearly 10,000 Swedish patients with MPNs, including about 4200 patients with PV, the risk of developing secondary non-hematologic malignancies was 60% higher than that observed in matched controls (standardized incidence ratio 1.4 for any non-hematologic malignancy and 3.3 for non-melanoma skin cancer); non-melanoma skin cancers, including SCC, BCC and Merkel cell carcinogenesis, were the most frequently reported malignancies (HR 2.8) [56]. In a cohort of 3941 patients with PV in the Unites States, the cumulative incidence of other primary malignancies was estimated to be 7.9% and 13.1% at 5 and 10 years, respectively, corresponding to an approximated standardized incidence ratio increase of 30% compared to that of the general population [59]. In a large real-world study based on the analysis of the data reported in Optimum Market Clarity electronic health record data on >20,000 PV US patients, 28% of patients developed secondary malignancies over 4.3 years of follow-up (9.6 cancers per 100 patient-years); the most common secondary malignancies were skin cancers (basal cell carcinoma, squamous cell carcinoma, and melanoma) [60]. The strongest risk factors for the occurrence of skin cancer included older age, previous history of skin cancer, and the use of cytoreductive medications, such as hydroxyurea [60]. Loscocco and coworkers reported the analysis of 1968 MPN patients (1001 PV and 967 with ET) with a median follow-up of 11.2 years. A history of another cancer was observed in 30% of patients and included cancers before/at diagnosis (15%) or after (20%) MPN diagnosis; patients who developed secondary cancer were significantly older at diagnosis compared with those who did not; secondary cancer was more frequent among patients with PV compared with ET patients (58% vs. 49%, respectively); cardiovascular risk factors, such as hypertension and hyperlipidemia, were more frequent in patients with secondary cancer; patients who developed secondary cancer had higher leukocyte counts and absolute neutrophil counts [57].

The pathogenic mechanisms underlying the higher risk of developing secondary cancer in PV remain in part unclear, and it was suggested that an intrinsic tendency toward malignancy, genetic predisposition, the effect of cytotoxic anti-neoplastic therapies, immune dysfunction and/or chronic inflammation could play a relevant role [61].

## 9. Molecular Determinants of Thrombosis

Thromboembolic events represent a consistent risk for PV patients and are a major cause of early mortality. The incidence of thrombotic events is age-dependent. PV patients left untreated display a high rate of death for thromboembolic events.

Thromboembolic events occurring after PV diagnosis may play a role in the risk of mortality, disease progression and occurrence of second cancer during follow-up [61,62,63,64,65]. A recent study evaluated the impact of thrombosis on disease progression, cancer and mortality in two cohorts of 3074 ET and 2302 PV patients [66]. In the PV cohort, 318 thromboembolic events were observed (181 arterial thrombosis and 137 venous thrombosis); patients displaying arterial thrombosis had significantly shorter OS compared to non-thrombosis patients, while no differences were observed according to venous thrombosis [60]. Similar findings were observed for ET patients. There was no significant association between thrombosis and progression to MF for both PV and ET patients [66]. In PV patients, thrombotic events were significantly associated with increased probability of secondary cancer: in particular, arterial thrombosis patients had a higher probability of secondary cancer, while venous thrombosis patients had only a trend towards higher probability of secondary cancer [66].

Many studies have attempted to identify patients who have a major risk for venous or arterial thrombosis, and among these, high *JAK2-V617F* burden emerged as a major determinant of the risk of developing venous thrombosis in PV patients [21,22]. Arterial thrombosis in PV patients seems to be more related to atherosclerosis and cardiovascular morbidities [21,22]. The causative link between high *JAK2-V617F* VAF and venous thrombosis seems to be related to the indirect effects of mutant *JAK2* on blood hyperviscosity and on endothelial dysfunction [21,22].

On the other hand, various non-driver mutations such as *ASXL1*, *TET2*, and *DNMT3A* seem to contribute to increasing the risk of thrombosis. Pasquer et al. have developed two prognostic models, one for arterial thrombosis (ARTS) and the other for venous thrombosis (VETS). The VETS score system evaluates the risk of venous thrombosis according to two criteria: previous history of venous thrombosis (1 point) and *JAK2-V617F* mutation with VAF ≥ 50% [67]. The ARTS scoring system evaluates the risk of arterial thrombosis according to four independent clinic-molecular factors: age at diagnosis > 60 years (2 points), prior arterial thrombosis (2 points), presence of *TET2* or *DNMT3A* mutations (1 point) and cardiovascular risk factors (smoking, hypertension, diabetes or hypercholesterolemia, 1 point) [67]. Based on cumulative points, patients are stratified into two primary risk tiers: low-risk (0–1 point) associated with an arterial thrombotic rate of 0.37% per patient-year; high-risk (2–6 points) associated with an arterial thrombotic event rate of 1.19% per patient-year; over a 10-year period, the probability of an arterial event is roughly 25% for high-risk patients compared to 6% for low-risk patients [67]. The ARTS performed better than two-tiered risk stratification, while the discrimination potential of VETS was limited and comparable to that of two-tiered conventional risk stratification [67].

*ASXL1*, *TET2* and *DNMT3A* mutations were significantly associated with arterial thrombotic events and marginally with venous events [44]. No association was found between thrombotic events and individual *ASXL1*, *DNMT3A* and *TET2* mutations; however, when considered for groups of two genes, the association with thrombotic events was significant for *TET2* with *DNMT3A* and *TET2* with *ASXL1*, but not with *DNMT3A* and *ASXL1*, suggesting that the association with thrombotic events was mainly mediated by mutations in *TET2* [44].

Recently, the European Leukemia Net (ELN) introduced a framework based on clinical signs and symptoms, including leukocytosis, splenomegaly, inadequate control, cardiovascular risk factors and severe pruritus, which showed predictive value for thrombosis across all risk groups, including low-risk patients [68]. A low but consistent risk of thrombotic events was also observed in low-risk polycythemia vera patients. In a recent study, based on the analysis of 437 LR-PV patients with a follow-up of 8.6 years, 17.4% of thrombotic events were observed (11.7% arterial and 5.7% venous). In these patients, thrombotic events are mainly driven by cardiovascular risk factors; cytoreductive therapy was associated with reduced thrombotic risk [69].

Recent studies have better defined that cardiovascular risk factors predict worse survival and thrombosis in PV patients but not progression to MF or to AML [70]. The QRISK3 score is a system used in the general UK population to measure cardiovascular risk, incorporating various clinical and demographic variables, such as age, history of hypertension, diabetes mellitus 2, atrial fibrillation, severe mental illness, and high BMI, to predict the risk of developing thrombotic events in PV and ET patients [71]. Media QRISK3 scores at diagnosis were higher in conventional high-risk PV and ET patients; during the follow-up of these patients, a QRISK3 score of >7.5% further stratified individuals at high risk of thrombotic events both in low-risk and high-risk groups of patients [71]. Importantly, cytoreductive therapy instead of active surveillance in patients with QRISK scores > 7.5% significantly reduced the risk of developing thrombotic events [71].

In conclusion, PV patients display a consistent risk of thrombotic events, arterial and venous, that represent a major cause of death in these patients. The risk of developing thrombotic events in these patients must be evaluated by clinical and biologic parameters, particularly in patients considered at high risk of this type of events. Both low-risk and high-risk PV patients must adopt therapeutic strategies reducing the risk of thrombotic events.

## 10. Current Clinical Applications

The considerable progress in understanding the mechanisms of the initiation, development and progression of PV modified and improved the multidisciplinary care approach to these patients. This background promoted the shift from symptom-focused to biology-focused treatments [72,73]. The main aims of PV treatment consist in obtaining a control of disease expansion and progression and in reducing the risk related to thrombotic events. PV management is based on an initial assessment at diagnosis of individual patients based on clinical parameters such as age and occurrence of thrombotic events; according to these criteria, the patients are subdivided into low-risk and high-risk. The primary objective in both low-risk and high-risk patients is to prevent thrombotic events through a control of hematocrit level and leukocyte number and the use of antiaggregant agents. The REVEAL study showed that both low-risk and high-risk PV patients’ maintenance of hematocrit level below 45% and WBC counts < 11 × 10^9^/L and platelets < 4 × 10^5^/μL are required to reduce the thrombotic risk [74]. Cytoreduction can be obtained in PV patients through phlebotomy or pharmacological treatments. Frequent phlebotomies may lead to iron deficiency, which can exacerbate PV patients’ symptoms. To bypass this limitation, an alternative approach consists in the use of drugs such as Rusfertide (approved in August 2026 by the FDA) that lower hematocrit through the hepcidin pathway by blocking iron absorption. Efficient pharmacological cytoreduction can be achieved in PV patients with three different types of drugs: hydroxyurea, a DNA synthesis inhibitor, recombinant alpha interferon or JAK 1-2 inhibitors. Recombinant IFN-alpha makes it possible to obtain a better disease control compared to hydroxyurea, and its use as a cytoreductive agent was also recommended in low-risk PV patients [75]. Recombinant alpha interferon and JAK 1-2 inhibitors offer the advantage, compared to hydroxyurea, that they can induce a reduction in *JAK2-V617F* burden and the achievement in some patients of molecular remission [75].

The treatment and management of PV patients is complex and must be individualized, considering the various clinical and biologic factors conditioning disease evolution and development of complications.

## 11. Conclusions

Studies carried out in recent years have provided a detailed molecular characterization of PV patients, supporting their consistent clinical and molecular heterogeneity. An emerging prognostic approach suggests that patients with PV need to be diagnosed and evaluated for their clinical and genomic profiles to define as well as possible their risk of thromboembolic events and of hematologic evolution. Each PV patient at diagnosis should be evaluated for clinical parameters (with particular emphasis on hematologic and cardiovascular evaluations), for genetic analysis to assess the presence of *JAK2-V617F*-mutant with VAF evaluation; in a subset of patients, particularly those at risk of progression or with atypical features, additional prognostic information may be obtained through analysis of non-driver mutations by targeted NGS and chromosomal abnormalities by standard cytogenetic analysis; this evaluation will provide criteria to elaborate an individual profile of risk of thromboembolic arterial and venous events and of hematologic evolution, and also to define personalized therapeutic options optimized to each patient [72].

## Data Availability

No new data were created or analyzed in this study. Data sharing is not applicable to this article.
